# Genome-scale methylation assessment did not identify prognostic biomarkers in oral tongue carcinomas

**DOI:** 10.1186/s13148-016-0235-0

**Published:** 2016-07-18

**Authors:** Annette M. Lim, Nicholas C. Wong, Ruth Pidsley, Elena Zotenko, June Corry, Alexander Dobrovic, Susan J. Clark, Danny Rischin, Benjamin Solomon

**Affiliations:** Department of Medical Oncology, Sir Charles Gairdner Hospital, Hospital Ave, Nedlands, Western Australia 6009 Australia; The University of Western Australia, Perth, Australia; Department of Paediatrics, The University of Melbourne, Parkville, Victoria 3010 Australia; Epigenetics Research Laboratory, Garvan Institute of Medical Research, 384 Victoria Street, Darlinghurst, New South Wales 2010 Australia; Department of Radiation Oncology, Peter MacCallum Cancer Centre, Victorian Comprehensive Cancer Centre Building, 305 Grattan St, Melbourne, Victoria 3000 Australia; The University of Melbourne, Melbourne, Australia; Translational Genomics and Epigenomics Laboratory, Olivia Newton-John Cancer Research Institute, 145 Studley Rd, Heidelberg, Victoria 3084 Australia; Department of Medical Oncology, Peter MacCallum Cancer Centre, Victorian Comprehensive Cancer Centre Building, 305 Grattan St, Melbourne, Victoria 3000 Australia; Department of Cancer Biology, La Trobe University, Bundoora, Victoria 3084 Australia

**Keywords:** DNA methylation, Tongue, Oral carcinoma, HM450K, Profiling, Survival, Epigenetics

## Abstract

**Background:**

DNA methylation profiling of heterogeneous head and neck squamous cell carcinoma (HNSCC) cohorts has been reported to predict patient outcome. We investigated if a prognostic DNA methylation profile could be found in tumour tissue from a single uniform subsite, the oral tongue. The methylation status of 109 comprehensively annotated oral tongue squamous cell carcinoma (OTSCC) formalin-fixed paraffin-embedded (FFPE) samples from a single institution were examined with the Illumina HumanMethylation450K (HM450K) array. Data pre-processing, quality control and analysis were performed using R packages. Probes mapping to SNPs, sex chromosomes and unreliable probes were accounted for prior to downstream analyses. The relationship between methylation and patient survival was examined using both agnostic approaches and feature selection. The cohort was enlarged by incorporation of 331 The Cancer Genome Atlas (TCGA) HNSCC samples, which included 91 TCGA OTSCC samples with HM450K and survival data available.

**Results:**

Given the use of FFPE-derived DNA, we defined different cohorts for separate analyses. Overall, similar results were found between cohorts. With an unsupervised approach, no distinct hypermethylated group of samples was identified and nor was a prognostic methylation profile identified. The use of multiple downstream feature selection approaches, including a linear models for microarray data (LIMMA), centroid feature selection (CFS), and recursive feature elimination (RFE) support vector machines, similarly failed to identify a significant methylation signature informative for patient prognosis or any clinicopathological data available. Furthermore, we were unable to confirm the prognostic methylation profiles or specific prognostic loci reported within the literature for HNSCC.

**Conclusions:**

With genome-scale assessment of DNA methylation using HM450K in one of the largest OTSCC cohorts to date, we were unable to identify a hypermethylated group of tumours or a prognostic methylation signature. This suggests that either DNA methylation in isolation is not likely to be of prognostic value or larger cohorts are required to identify such a biomarker for OTSCC.

**Electronic supplementary material:**

The online version of this article (doi:10.1186/s13148-016-0235-0) contains supplementary material, which is available to authorized users.

## Background

One of the characteristic epigenetic hallmarks of cancer is aberrant DNA methylation, the presence of a 5′-methyl group on cytosine bases in the context of CpG dinucleotides. Across numerous malignancies, both aberrant global hypomethylation and promoter hypermethylation of numerous loci have been reported to promote carcinogenesis by increasing genomic instability and by altering gene expression [1–4]. In cancer, epimutations including promoter DNA hypermethylation, are known to occur more frequently than genomic abnormalities [5], and thus form an attractive target for biomarker discovery. Furthermore, the identification of CpG island methylator phenotypes (CIMP) in glioblastomas and colorectal carcinomas have provided proof-of-principle that methylation profiling could provide a means to identify subsets of epigenetically and genetically distinct diseases that are informative for patient outcome [6, 7].

In head and neck squamous cell carcinomas (HNSCC), both locus-specific methylation and methylation profiles identified with a variety of methods have been reported to be both biologically and prognostically significant [8–14]. However, the clinical utility of these reported hypermethylated loci has been limited by the use of heterogeneous HNSCC cohorts and is further complicated by the diversity of non-reproduced loci identified by these reports [15]. Little is understood about the significance of methylation according to individual head and neck subsites, including oral tongue squamous cell carcinomas (OTSCC), which have the worst overall survival for early-stage disease [16, 17]. Beyond the subset of human papillomavirus-induced oropharyngeal carcinomas [18, 19], no clinically impacting molecular markers are utilised routinely for all other head and neck cancers. Therefore, the burden of need exists for the identification of clinically relevant biomarkers to assist with improved disease stratification and treatment allocation.

The Illumina HumanMethylation450K (HM450K) array provides a quantitative high throughput platform for the genome-scale assessment of methylation [20]. This array interrogates a total of 482,421 CpG sites located predominantly in promoter regions, but crucially also includes coverage of CpG island (CGI) shores which contain the most differentially methylated regions in cancer [2, 21]. Thus, in comparison to a candidate gene approach, CpG sites can be simultaneously interrogated for the purpose of identifying potentially informative specific loci or methylation signatures.

We sought to identify a clinically relevant methylation profile in a large cohort of comprehensively annotated OTSCC with the HM450K array. In addition, methylation data from the heterogeneous cohort of HNSCC samples generated by The Cancer Genome Atlas (TCGA; http://cancergenome.nih.gov/) were analysed, which included a similar sized subset of OTSCC.

## Results

### Patient samples

The OTSCC patient cohort from the Peter MacCallum Cancer Centre has been previously described (Table 1) [15, 22]. Briefly, the cohort consisted of patients with invasive OTSCC, with comprehensive clinicopathological details and pre-treatment archival specimen blocks. For this study, 109/131 samples had sufficient DNA for analysis on the HM450K array.

**Table 1:** Patient and tumour characteristics [22]

	Number	Percent
Gender		
Female	48	37
Male	83	63
Age at diagnosis (in years)		
Mean	57.8	
Standard deviation	15.1	
Median	60	
Range	21–93	
<40 years	15	11
40–49 years	26	20
50–59 years	24	18
60–69 years	32	24
70–79 years	27	21
80+ years	7	5
Stage		
1	37	28
2	37	28
3	15	11
4	42	32
T category		
1	43	33
2	51	39
3	14	11
4	23	18
N category		
0	83	63
1	14	11
2	34	26
Smoking history		
No/never	39	30
Yes	88	67
Unknown	4	
Alcohol >20 g/day		
No/never/social	81	62
Past or current	46	35
Unknown	4	
ECOG performance status		
0	76	58
1	40	31
2	9	7
3	4	3
Unknown	2	

Level 1 raw IDAT files were downloaded from the TCGA data portal (https://tcga-data.nci.nih.gov) on 24 June, 2013, and the clinical annotation was downloaded on 22 July 2013. The data we used was a superset of data used in the published TCGA head and neck cancer analysis [23]. For our analyses, the TCGA HNSCC cohort consisted of 373 patient samples with HM450K data available. Only limited clinical annotation was available for these TCGA samples, thus only the variables of overall survival status and tumour-node-metastases (TNM) stage were used for downstream analyses. Patients with metastatic disease or those with an unknown M category (whereby the M category indicates the presence of metastatic disease) were excluded from further analysis, leaving a cohort of 331 TCGA HNSCC samples, which included 91 OTSCC. The samples used are listed according to anatomical subsite in Additional file 1: Table S1. Additional file 2 lists the downloaded TCGA sample IDs, the samples included for analysis and those also analysed in the TCGA head and neck cancer analysis [23].

The data was grouped for interrogation in three ways. The first group consisted of 109 OTSCC samples processed in one batch from the Peter MacCallum Cancer Centre (“OTSCC cohort”). The second group combined the pre-processed OTSCC cohort with the 91 OTSCC samples from the TCGA database, which we termed the “combined OTSCC cohort”. The final analysis was conducted on the “entire cohort” of samples, comprising 109 OTSCC and all 331 TCGA HNSCC samples (total *n* = 440).

### Data analyses

**Fig. 1:**
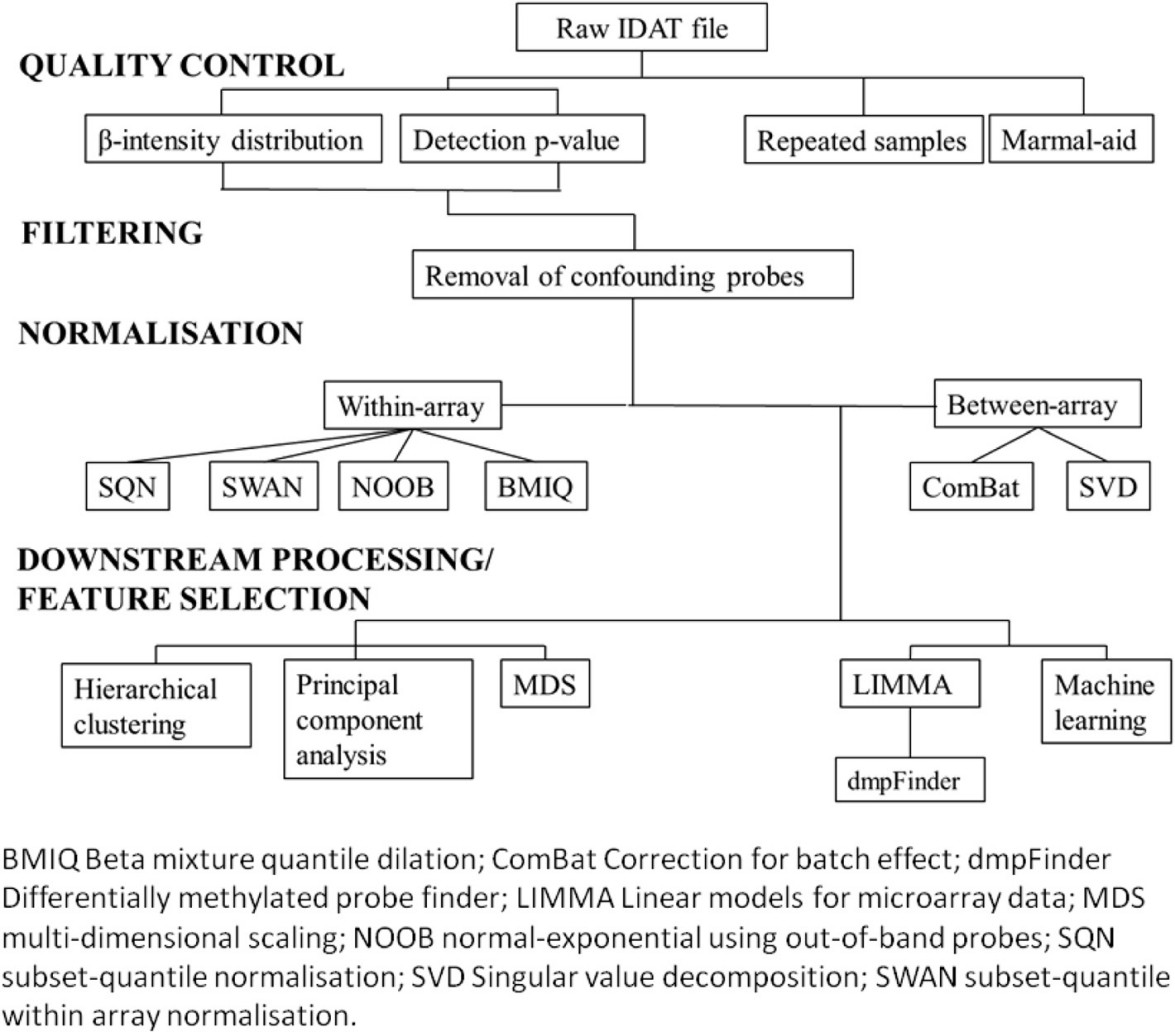
Workflow for bioinformatics processing of raw data

Bioinformatics processing of the raw IDAT files was performed utilising R statistical software (version 3.0.1, http://cran.r-project.org/). Currently, no consensus guidelines exist on the optimal method of pre-processing HM450K or the optimal method of feature selection. Given the variety of methods accounting for the same technical biases without clear indication of superiority between the techniques, for the pre-processing and subsequent downstream analyses described below, each method was considered of similar quality. The workflow for the bioinformatics analysis is summarised in Fig. 1, and the methods of analysis for each of the cohorts is summarised in Additional file 1: Table S2, according to the R software library packages utilised.

### Data pre-processing, filtering and normalisation of data

Due to the different pre-processing criteria and stringency criteria utilised according to each R package, different subgroups from each cohort were defined and were analysed separately. Details of the different criteria utilised according to each library is outlined in the “Methods” section and in Additional file 1: Table S2. However, regardless of what pre-processing criteria were utilised, the size of the refined cohort examined or the combinations of cohorts analysed, results for the downstream analyses were similar.

### Downstream processing and feature selection

#### Unsupervised feature selection

**Fig. 2:**
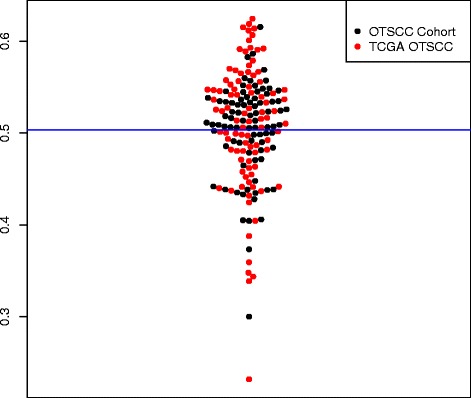
Plot of median beta values of the combined OTSCC cohort. A plot of the median beta values for the combined cohort of 174 OTSCC samples with outcome data, with the *blue line* indicating the overall median value for the group. A bimodal distribution is observed, without evidence of a hypermethylated group of samples

**Fig. 3:**
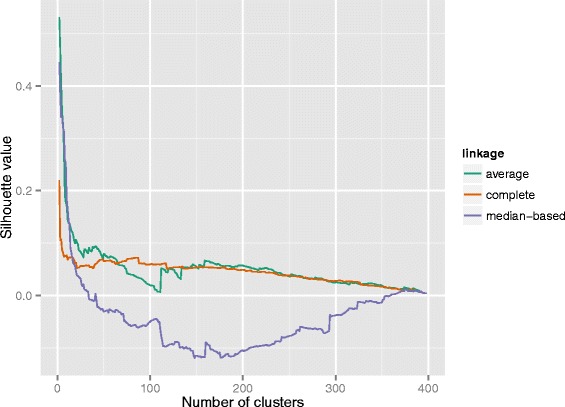
Plot of the average silhouette value according to the number of clusters. This was assessed on RnBeads using an average linkage-based algorithm. The combined cohort demonstrates no distinct clustering according to observed methylation values. A silhouette value of “1” indicates good classification of the observation into the cluster, a value of “0” indicates that the observation lies independent of the groupings, whilst a negative value indicates poor classification and likely incorrect grouping

After quality control and pre-processing of 450K data using Minfi and Methylumi, 83/109 samples from the OTSCC cohort, 174 (83 OTSCC cohort plus 91 TCGA OTSCC) from the combined cohort and a total of 414 samples for the entire cohort were taken through to analysis. A bimodal distribution of *β* values for the OTSCC cohort and combined OTSCC cohort was observed indicating the absence of a group of samples with promoter hypermethylation (Fig. 2). Similarly, hierarchical clustering, multidimensional scaling (MDS) plots and principal components analysis did not clearly identify a subgroup of differentially methylated samples according to phenotypic data (Additional file 1: Figure S1). These findings were again confirmed using another technique in RnBeads to look at the clustering of data, mean silhouette values. Figure 3 demonstrates the poor classification of samples using this method and the numerous small clusters identifiable throughout the dataset, with little similarities between them. A silhouette value of “1” indicates good classification of the observation into the cluster, and a value of “0” indicates that the observation lies independent of the groupings, whilst a negative value indicates poor classification and likely incorrect grouping (http://stat.ethz.ch/R-manual/R-devel/library/cluster/html/silhouette.html) [24]. Given that increasing the number of clusters makes the similarities within the cluster worse (approaches “0”), this suggests that the clustering of samples is based on very subtle and small differences in methylation.

Therefore, despite increasing the OTSCC cohort size by inclusion of the 91 TCGA OTSCC samples to create a combined cohort of 174 OTSCC samples, DNA methylation data did not identify any unique subgroups according to phenotypic data. Investigating the entire cohort of 414 heterogeneous HNSCC samples also failed to demonstrate clear clustering of samples according to methylation profiles (Additional file 1: Figure S1). This was not surprising as heterogeneous cohorts of head and neck samples are likely to demonstrate heterogeneous methylation profiles.

#### Feature selection methods

Utilising a variety of feature selection methods, we sought to determine if methylation was informative for patient survival or other clinicopathological variables.

For the OTSCC cohort, the relationship between DNA methylation and multiple phenotypic variables was investigated. This included assessment of relationship with overall survival, smoking history, history of alcohol excess and age <45 years versus >45 years due to the recognition of a subpopulation of younger patients that develop OTSCC in the absence of any risk factors [25, 26]. Of particular interest was to determine if tumours with nodal involvement had a differential methylation profile compared to those without, given that nodal status (N category) is one of the most reliable clinical risk classifiers of worse prognosis [27]. Analyses investigating the relationship between DNA methylation and ECOG performance status, progression-free survival, disease-specific survival, T category, pathological differentiation status, and gender were also performed.

Based on the clinical annotation available for the TCGA cohort, the supervised analyses for the combined OTSCC cohort and the entire cohort only investigated the association between DNA methylation and overall survival status, and DNA methylation and TNM staging.

##### Linear models for microarray data analyses

A linear models for microarray data (LIMMA) framework was initially utilised to interrogate the pre-processed OTSCC cohort (*n* = 83). After correction for multiple testing, significant associations between DNA methylation and gender, age and pathological differentiation status were identified (*q* values <0.05, Benjamini-Hoechberg method). These specific variables were investigated further to see if they were informative for patient survival. Given the small numbers of samples in the age less than 45 years category (less than 10 samples), only methylation status according to gender and differentiation status were investigated for their impact on patient outcome.

For the analysis according to differentiation status, there were 25 patients with poorly differentiated tumours compared with 58 well differentiated tumours. Of these poorly differentiated tumours, 13 patients died. For the analysis according to gender, there were 57 male patients and 26 female patients, of which 27 male and 11 female patients survived. The LIMMA framework was used to discern if probes that significantly defined a variable were also associated with patient survival. However, no significant association between a tumour’s differentiation status stratified according to differential methylation profiles, and patient survival was found (*q* value >0.05). Similarly, no association was found when analysed according to patient gender (*q* value >0.05).

When the combined OTSCC cohort (*n* = 174) was investigated with dmpFinder, after correction for multiple testing, methylation values were found to be uninformative for survival status and TNM stage. For overall survival, 28,078 probes had a *p* value of less than 0.05 but failed to reach significance (*q* value >0.05) after correction for multiple testing. When disease stage was used as the classifier, 19,472 probes were identified with *p* values <0.05, with one probe remaining after correction for multiple testing. This single probe within a promoter region corresponded to a SNP (cg17508434, a promoter-associated CpG to the LMTK2 gene). Please refer to the “Methods” section for information regarding filtering of probes. In a separate analysis where filtering of SNP-associated probes were removed, no informative probes were identified.

Therefore, these analyses suggested that methylation values did not inform patient outcome. The small numbers of events as described may have impacted these findings.

##### Machine learning approaches

Machine learning algorithms were also employed for analyses of the data. These were wrapped into a tool called “Genome toolbox” (GTB), a custom R library (personal communication Dr Justin Bedo, IBM) [28, 29]. Initially, the OTSCC cohort (*n* = 83) was assessed using centroid feature selection (CFS) with three-fold cross validation. A performance of 1.0 represents a 100 % chance of being able to classify groups according to the nominated variable, according to the number of methylation probes interrogated. A prediction performance of 0.5 represents a prediction capability no better than chance to classify the variable. Overall, prediction performance was poor and ranged between 0.5 to 0.7 for classification of pathological differentiation status, history of alcohol excess and smoking history according to DNA methylation. The prediction performance utilising other phenotypic variables was worse. The top eight ranked methylation probes identified to be informative for overall survival in the OTSCC cohort (cg141693, cg079784, cg148610, cg146787, cg184013, cg080496, cg137586, cg0322520) failed to classify samples according to survival status in the combined OTSCC cohort and the entire cohort. When the alternate method of recursive feature elimination – support vector machine (RFE-SVM) was used, again no significant association between DNA methylation and survival status was identified.

**Fig. 4:**
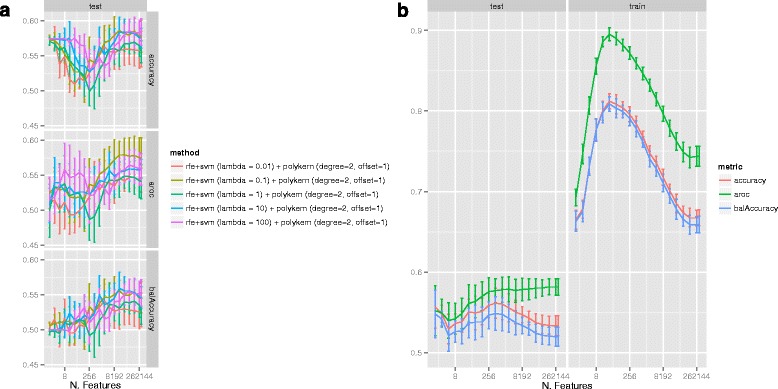
Prediction performance plots for overall survival. **a** Prediction performance plot for overall survival of the combined oral tongue cohort of the mean value derived from the machine learning method RFE-SVM using a range of parameters (lambda range 0.01 with a poly kernel). *Error bars* represent the range of values obtained across the three-fold cross validation performed. The prediction performance ranged between 0.5 and 0.58 indicating a poor performance. **b** Representative prediction performance plot for overall survival of the combined oral tongue cohort using CFS. The training set demonstrates a decrease in performance with an increasing number of probes utilised, and the test model derived from the training set shows poor performance. *Error bars* represent the range of values across the three-fold cross validation performed. For both plots, a performance of 1.0 represents a 100 % chance of being able to classify groups according to the nominated variable, according to the number of probes interrogated. A prediction performance of 0.5 represents a capability of no better than chance

A similar analysis was performed using machine learning methods for the larger combined OTSCC cohort (*n* = 174), using overall survival status as the classifying variable. For each method, ranges of tuning parameters (kernels and lambda values) were employed to optimise the prediction performance. However, both CFS and RFE-SVM models demonstrated no prediction capability, with performance ranging from 0.52 to 0.58 for both methods (whereby a prediction performance of 0.5 indicates no prediction capability beyond chance, Fig. 4).

In addition, using these machine learning frameworks, a variety of other mathematical models were investigated including the *t* test and the LIMMA method. These analyses similarly demonstrated poor prediction performance metrics supportive of the conclusion that a prognostic methylation signature did not exist for the cohort.

## Discussion

In a uniform cohort of samples from a single head and neck subsite, using both an agnostic approach to the data and a number of feature selection approaches, we were unable to identify a differentially methylated group of samples and we were unable to identify a methylation profile informative for clinicopathological features.

A number of previous reports have suggested the identification of prognostic signature for HNSCC, using a variety of approaches including high throughput methylation assessments [8, 11–13, 30–32]. However, the cohorts examined were generally small, samples analysed were from heterogeneous head and neck subsites, and though some groups identified a hypermethylated group of samples and prognostically informative loci, none of these were reproducible between studies. Furthermore, some prognostically significant probes identified correspond to known SNPs [8, 11], which raises concerns regarding the reliability of these probes to assay DNA methylation. Our study of a large cohort of samples from a single subsite was unable to identify a hypermethylated group of samples or identify an informative methylation signature. Interestingly, the TCGA analyses of a heterogeneous group of 279 HNSCC samples that overlapped the samples we utilised (Additional file 2) identified four groups of tumours according to DNA methylation status, with a hypermethylated, hypomethylated, normal-like and CpG island methylated group of samples [23]. However, the association between methylation group and survival status was not significant ([23], Supplementary Figure S1.5b, *p* = 0.05).

Technical issues relating to the HM450K platform itself may have impaired the identification of a methylation profile. The use of formalin-fixed paraffin-embedded (FFPE)-derived DNA for the OTSCC cohort may have affected findings by affecting the quality of reported methylation or the number of CpG sites assessed. In part, this was accounted for by the use of different stringency filtering criteria. In recognition of the fact that an allelic state (SNP) may be informative for the methylation status of particular loci and may correlate with disease states [33, 34], analyses were performed that included and excluded all SNP probes. However, no differences in results were observed. Perhaps the most significant limitation of the HM450K array is that it only interrogates ~2 % of CpGs within the human genome, and thus informative regions may be missed. At present, there are a limited number of whole genome bisulfite sequencing studies, with only one cancer-related study published which investigated a single colorectal cancer sample [35]. Our findings may also have been limited by the sample size of the cohort. However, this study represents one of the largest of its kind that examines a single head and neck subsite with high throughput methodology.

We tested whether observed methylation in isolation served as a prognostic marker or whether it correlated with any clinicopathological feature. However, the clinical impact of altered methylation is likely to be influenced by other factors that this approach does not account for. These other factors include whether the quantity of detected methylation is relevant, the location of informative CpG sites, and the known complex interactions between DNA methylation and other genetic and epigenetic factors, particularly those regulated by the polycomb repressor group proteins [5, 36, 37].

The relationship between DNA methylation and gene expression is complex, given the absence of a uniform effect of the presence of methylation. This impacts the findings of correlative analyses. Different threshold quantities of methylation has been found to differentially impact gene expression according to CpG location and tissue histotype in a study by Liu et al. which examined 992 different tumour samples from the TCGA project assessed by high throughput methylation analyses [38]. Similarly, though the functional impact of hypermethylation of CGIs in promoter regions is best understood and is thought to usually correlate with gene repression [5], the location of informative CpG sites is not comprehensively known. Increasingly, research demonstrates that methylation outside promoter regions is also informative and that the presence of methylation does not always correlate with gene repression [39]. Similarly, long-range epigenetic activation (LREA) and long-range epigenetic silencing (LRES) needs to be considered, which refers to the regional activation or silencing of often very large domains that can be mediated by DNA hypermethylation [35, 40]. LRES and LREA may result in the differential expression of genes, through the induction of a DNA conformational change enlisting the use of alternate transcription start sites or through the inhibition of transcription repression factors, similar to that seen with the CTCF insulator proteins [39–41]. Lastly, an analysis of the clinical significance of methylation in isolation ignores the complex interplay between the genome and epigenome. This is easily demonstrated through the known significant clinical impact of the CIMP-high phenotype in colorectal cancer and the simultaneous presence of *TP53* mutations and *BRAFV600E* mutations or, similarly, the G-CIMP subgroup of glioma tumours with concurrent *IDH1* mutations [6, 7]. Indeed, the TCGA analysis of 279 heterogeneous HNSCC identified a significant relationship between hypomethylation and the loss of function mutation of *NSD1*, but the impact on patient outcome was not reported [23]. The interaction between DNA methylation and other epigenetic modifiers, such as histone modifications, is also complicated by a possible bivalent state of gene transcription, where both active marks such as acetylation (e.g. H3K9ac), and repressive marks such as H3K27me3, can associate with DNA methylation [5, 37, 40, 42]. This indicates that the presence of DNA methylation is not necessarily representative of a binary effect on gene expression and thus its correlation with clinicopathological features.

Our analysis sought to determine whether methylation in isolation heralded any prognostic information for OTSCC patients, and to some extent, the approach did account for some of the more complex issues discussed. The agnostic analyses with the unsupervised feature selection avoided assumptions made on the significance of *β* values, the location of informative probes or even the resultant impact of altered methylation. However, it may be that methylated regions defined according to the effect on gene expression may need to be identified before reliable clinical correlations will be observed.

## Conclusions

The detailed assessment of the methylation status of over 450,000 CpG sites with the HM450K array in a comprehensively annotated OTSCC cohort importantly demonstrated that a methylation signature or prognostically informative CpG sites could not be found. The presence of DNA methylation could not stratify clinicopathological variables according to impact on patient outcome. Although a number of technical factors have been discussed, the absence of an independent prognostic methylation profile likely results from the complex and dynamic interaction of DNA methylation with other genomic and epigenomic mechanisms of gene regulation. Whole genome bisulfite sequencing in combination with other “omic” profiling may provide additional information regarding the biological impact of methylation. However, within the limitations of our current understanding, the use of FFPE-derived DNA, and the size of the cohort examined, our findings suggest that methylation alone is unlikely to inform patient outcome for OTSCC.

## Methods

### Preparation of bisulfite-modified DNA

DNA extraction and bisulfite modification has been previously described [15, 22]. One microgram of DNA was bisulfite modified (BS) and eluted twice, into a total volume of 100 μL. DNA precipitation was utilised to prepare the BS DNA for a final volume of 7 μL resuspended in TE buffer for processing on the HM450K array. DNA quality post bisulfite modification and precipitation was confirmed by checking the known *CDKN2A* methylation status by MS-HRM on selected samples.

### Methylation analysis

Samples were sent to the Australian Genome Research Facility (AGRF, Melbourne, Australia) for processing on the HM450K array as a single batch. Methodology for the analysis has been previously published [43, 44]. Raw IDAT files of the data generated were provided to us by AGRF. Data from samples passing initial quality filtering have been deposited in GEO (Gene Expression Ominibus) GSE79556.

### Bioinformatics analysis

Given the absence of consensus on processing of high throughput methylation data, we chose to approach the data as comprehensively as possible with different software libraries and packages (Additional file 1: Table S2). The aim of this approach was to internally validate findings with orthogonal methodology or similar methodology but also to optimise opportunity for identifying significant correlations. Separate analyses were performed using each package to pre-process data, and for each refined cohort, downstream analyses were performed as described below.

Bioinformatics processing of the raw IDAT files was performed utilising R statistical software (version 3.0.1, http://cran.r-project.org/). Software library packages used included Methylumi version 2.6.1 (http://www.bioconductor.org/packages/release/bioc/html/methylumi.html), Minfi version 1.6.0 (http://www.bioconductor.org/packages/2.12/bioc/html/minfi.html), ChAMP version 0.99.0 (http://www.bioconductor.org/packages/2.13/bioc/html/ChAMP.html) [45], wateRmelon version 1.0.3 (http://www.bioconductor.org/packages/2.13/bioc/html/wateRmelon.html) [46], RnBeads version 0.99.9 (http://rnbeads.mpi-inf.mpg.de/) and a custom machine learning package (GTB version 0.0.1) [28, 29] (personal communication Dr Justin Bedo, IBM).

### Pre-processing of data

We performed standard quality control assessments in-built into the library packages utilised. The raw data initially underwent quality control assessment through inspection of the *β* value distribution plots, for the identification of outlying samples. Detection *p* values were then used to identify failed probes defined as those that did not emit a signal above background levels [45, 46]. In addition, two further quality assurance measures were performed prior to downstream analysis; Firstly, our data was found to be comparable and did not lie outside of other HM450K datasets, by analysis of the cohort with 8654 publically available samples curated on Marmal-aid (http://marmal-aid.org/, version 1.1.1) [47] (Additional file 3). Secondly, a number of samples were also processed and analysed in replicate on the HM450K array by AGRF, which confirmed the reproducibility of results (data not shown). We have previously published results from this cohort that compared the methylation analyses of specific loci utilising four orthogonal methodologies (HM450K array, SMART-MSP, pyrosequencing and MS-HRM) with the TCGA HNSCC dataset, which confirmed the reproducibility and robustness our data [15].

### Filtering of the data

As there is no consensus on which probes should be removed from downstream analysis, a variety of filtering criteria was used to account for the issues described further (summarised on Additional file 1: Table S2), thereby refining different sized cohorts. Despite the different sizes of the cohorts refined from the use of different criteria, including and excluding SNPs, overall, downstream analyses for these different cohorts created by the use of different filtering and normalisation methods were similar. Factors accounted for in our approach to the filtering of data are described further below.

The HM450K was initially reported to include 3091 probes interrogating non-CpG sites (context specific probes), and 65 single nucleotide polymorphisms (SNPs) [20]. However, in addition to these, it is recognised that confounding of results may be introduced by the following: an additional (unreported) 66,877 SNP probes, at or in very close proximity to the interrogated CpG site; by the sex chromosome probes; by consistently poor performing probes; and by co-localising (off-target mapping) probes [48–52]. Whilst most studies have determined the number of confounding probes using in silico methods, when HM450K data was compared with data generated by whole genome bisulfite sequencing, up to approximately 200,000 probes were found to introduce artefactual methylation assessments and require consideration of removal prior to downstream processing [52]. The sex chromosome probes introduce bias as X-inactivation is regulated by hypermethylation, and the copy number of X-chromosomes varies according to gender [49]. This is additionally relevant for autosomal probes that hybridise to the sex chromosomes. Some probes reporting high intensity *β* values are noted to be unreliable on repeated analyses, whereby methylation values are unable to be replicated by alternative method [48]. In addition, up to 9 % (~42,000) probes are reported to co-localise to alternative sites due to homologous sequences elsewhere, such as pseudogenes [50]. Dependent on the choice of alignment stringency (for example, the number of mismatches permitted), one study determined that up to 29 % (~140,000) probes misaligned when two mismatches were permitted [51]. Probes interrogating SNPs may introduce artefactual methylation measurements dependent on the genotype of the patient and the frequency of the polymorphism in the population. Furthermore, SNPs may also interfere with probe hybridisation dynamics if a SNP exists within the probe target sequence. This may alter the observed methylation measurements from the true extent of methylation at these sites [48, 49]. However, specific allelic states (SNPs) are recognised to be significantly associated with the methylation status of certain genes [33, 34], which argues that it may be crucial to include SNP probes in downstream processing.

### Data normalisation

Background fluorescence correction was achieved through methods available through Methylumi and by utilising the normal exponential using out-of-band probes (NOOB) method. Data normalisation was also performed to account for the significant technical biases that result from the use of different chemistry underlying the two probes on the HM450K array, with the Infinium II probes demonstrating a smaller dynamic range with less sensitivity for methylation values at extreme ranges and inferior reproducibility of methylation values with repeated testing [53]. These biases were accounted for within the analysis using the subset-quantile normalisation (SQN) methods available through Methylumi and Minfi packages [54], subset-quantile within array normalisation (SWAN) [44], and beta mixture quantile dilation (BMIQ) [55, 56].

Batch variation was corrected using the singular value decomposition (SVD) method [57], and the ComBat method [58] available on the ChAMP library. A customised method of pre-processing the data was also performed using the wateRmelon package, using the “dasen” method of normalisation of β-intensities of each probe type [46].

### Downstream processing and feature selection

For the agnostic interrogation of data, unsupervised feature selection was performed using multidimensional scaling (MDS) plots, hierarchical clustering, and principal components analysis. Two main supervised feature selection approaches were used to identify differential methylation. Firstly, a linear models for microarray data (LIMMA) approach was used, through “dmpFinder” (differentially methylated probe Finder) with false discovery rates (FDR) of a *p* value less than 0.05 [59, 60]. RnBeads also hosts a method of assessing methylation according to specified regions (compared to a single probe). Secondly, machine learning methods were also used which were based on a recursive feature elimination (RFE) framework and incorporated recursive feature elimination – support vector machine (RFE-SVM), as well as centroid feature selection (CFS). Both methods used a three-fold cross validation [28]. The machine learning approach to supervised feature selection is an empirical method to analyse the data in its entirety, where training and learning models to select a nominated feature are generated by cross validation within the dataset.

## Abbreviations

BMIQ, beta mixture quantile dilation; BS, bisulfite modified; CFS, centroid feature selection; ComBat, correction for batch effect; dmpFinder, differentially methylated probe finder; ECOG, Eastern Cooperative Oncology Group; HNSCC, head and neck squamous cell carcinomas; LIMMA, linear models for microarray data; MDS, multi-dimensional scaling; NOOB, normal-exponential using out-of-band probes; OTSCC, oral tongue squamous cell carcinomas; RFE-SVM, recursive feature elimination – support vector machine; SNPs, single nucleotide polymorphisms; SQN, subset-quantile normalisation; SVD, singular value decomposition; SWAN, subset-quantile within array normalisation; TCGA, The Cancer Genome Atlas; TNM, tumour-node-metastases.

## Additional files

Additional file 1: Supplementary figures and tables. Figure S1. Unsupervised clustering of analysis of OTSCC cohort and combined OTSCC cohort of samples. A) MDS plot of the 1000 most variable methylation values from the OTSCC cohort demonstrating no distinct groups. B) Dendrogram of methylation values from OTSCC cohort demonstrating at least seven distinct subgroups. C) Dendrogram of methylation values including good quality SNP probes, from the entire cohort of 414 samples, demonstrating at least 15 subgroups. Table S1. The TCGA HNSCC cohort with sample numbers listed according to anatomical subsite. Table S2. Summary of bioinformatics processing of the different cohorts of input data. (DOC 525 kb) Additional file 2: CSV file listing the TCGA samples utilised in our analysis and in the TCGA head and neck cancer analysis [23]. (CSV 10 kb) Additional file 3: Summary of additional quality assurance analyses of our dataset against other public datasets. (DOCX 733 kb)

## References

[1] Poage GM, Houseman EA, Christensen BC, Butler RA, Avissar-Whiting M, McClean MD, Waterboer T, Pawlita M, Marsit CJ, Kelsey KT (2011). Global hypomethylation identifies loci targeted for hypermethylation in head and neck cancer. Clin Cancer Res.

[2] Irizarry RA, Ladd-Acosta C, Wen B, Wu Z, Montano C, Onyango P, Cui H, Gabo K, Rongione M, Webster M (2009). The human colon cancer methylome shows similar hypo- and hypermethylation at conserved tissue-specific CpG island shores. Nat Genet.

[3] Lawrence MS, Stojanov P, Polak P, Kryukov GV, Cibulskis K, Sivachenko A, Carter SL, Stewart C, Mermel CH, Roberts SA (2013). Mutational heterogeneity in cancer and the search for new cancer-associated genes. Nature.

[4] Gaudet F, Hodgson JG, Eden A, Jackson-Grusby L, Dausman J, Gray JW, Leonhardt H, Jaenisch R (2003). Induction of tumors in mice by genomic hypomethylation. Science.

[5] Baylin SB, Jones PA (2011). A decade of exploring the cancer epigenome—biological and translational implications. Nat Rev Cancer.

[6] Noushmehr H, Weisenberger DJ, Diefes K, Phillips HS, Pujara K, Berman BP, Pan F, Pelloski CE, Sulman EP, Bhat KP (2010). Identification of a CpG island methylator phenotype that defines a distinct subgroup of glioma. Cancer Cell.

[7] Hinoue T, Weisenberger DJ, Lange CP, Shen H, Byun HM, Van Den Berg D, Malik S, Pan F, Noushmehr H, van Dijk CM (2012). Genome-scale analysis of aberrant DNA methylation in colorectal cancer. Genome Res.

[8] Poage GM, Butler RA, Houseman EA, McClean MD, Nelson HH, Christensen BC, Marsit CJ, Kelsey KT (2012). Identification of an epigenetic profile classifier that is associated with survival in head and neck cancer. Cancer Res.

[9] Ai L, Vo QN, Zuo C, Li L, Ling W, Suen JY, Hanna E, Brown KD, Fan CY (2004). Ataxia-telangiectasia-mutated (ATM) gene in head and neck squamous cell carcinoma: promoter hypermethylation with clinical correlation in 100 cases. Cancer Epidemiol Biomarkers Prev.

[10] Roh JL, Wang XV, Manola J, Sidransky D, Forastiere AA, Koch WM (2013). Clinical correlates of promoter hypermethylation of four target genes in head and neck cancer: a cooperative group correlative study. Clin Cancer Res.

[11] Jung AC, Job S, Ledrappier S, Macabre C, Abecassis J, de Reynies A, Wasylyk B (2013). A poor prognosis subtype of HNSCC is consistently observed across methylome, transcriptome, and miRNome analysis. Clin Cancer Res.

[12] Lechner M, Fenton T, West J, Wilson G, Feber A, Henderson S, Thirlwell C, Dibra HK, Jay A, Butcher L (2013). Identification and functional validation of HPV-mediated hypermethylation in head and neck squamous cell carcinoma. Genome Med.

[13] Jithesh PV, Risk JM, Schache AG, Dhanda J, Lane B, Liloglou T, Shaw RJ (2013). The epigenetic landscape of oral squamous cell carcinoma. Br J Cancer.

[14] Chen LH, Liu DW, Chang JL, Chen PR, Hsu LP, Lin HY, Chou YF, Lee CF, Yang MC, Wen YH (2015). Methylation status of insulin-like growth factor-binding protein 7 concurs with the malignance of oral tongue cancer. J Exp Clin Cancer Res.

[15] Lim A, Candiloro I, Wong N, Collins M, Do H, Takano E, Angel C, Young R, Corry J, Wiesenfeld D (2014). Quantitative methodology is critical for assessing DNA methylation and impacts on correlation with patient outcome. Clin Epigenetics.

[16] Rusthoven K, Ballonoff A, Raben D, Chen C (2008). Poor prognosis in patients with stage I and II oral tongue squamous cell carcinoma. Cancer.

[17] Brennan S, Corry J, Kleid S, Porceddu S, Yuen K, Rischin D, Peters LJ (2010). Prospective trial to evaluate staged neck dissection or elective neck radiotherapy in patients with CT-staged T1-2N0 squamous cell carcinoma of the oral tongue. Head Neck.

[18] Rischin D, Young RJ, Fisher R, Fox SB, Le QT, Peters LJ, Solomon B, Choi J, O'Sullivan B, Kenny LM, McArthur GA (2010). Prognostic significance of p16INK4A and human papillomavirus in patients with oropharyngeal cancer treated on TROG 02.02 phase III trial. J Clin Oncol.

[19] Gillison ML, D'Souza G, Westra W, Sugar E, Xiao W, Begum S, Viscidi R (2008). Distinct risk factor profiles for human papillomavirus type 16-positive and human papillomavirus type 16-negative head and neck cancers. J Natl Cancer Inst.

[20] Bibikova M, Barnes B, Tsan C, Ho V, Klotzle B, Le JM, Delano D, Zhang L, Schroth GP, Gunderson KL (2011). High density DNA methylation array with single CpG site resolution. Genomics.

[21] Sandoval J, Heyn H, Moran S, Serra-Musach J, Pujana MA, Bibikova M, Esteller M (2011). Validation of a DNA methylation microarray for 450,000 CpG sites in the human genome. Epigenetics.

[22] Lim AM, Do H, Young RJ, Wong SQ, Angel C, Collins M, Takano EA, Corry J, Wiesenfeld D, Kleid S (2014). Differential mechanisms of CDKN2A (p16) alteration in oral tongue squamous cell carcinomas and correlation with patient outcome. Int J Cancer.

[23] Cancer Genome Atlas N (2015). Comprehensive genomic characterization of head and neck squamous cell carcinomas. Nature.

[24] Rousseeuw PJ (1987). Silhouettes: a graphical aid to the interpretation and validation of cluster analysis. J Comput Appl Math.

[25] Hilly O, Shkedy Y, Hod R, Soudry E, Mizrachi A, Hamzany Y, Bachar G, Shpitzer T (2013). Carcinoma of the oral tongue in patients younger than 30 years: comparison with patients older than 60 years. Oral Oncol.

[26] Toner M, O'Regan EM (2009). Head and neck squamous cell carcinoma in the young: a spectrum or a distinct group? Part 1. Head Neck Pathol.

[27] Goldstein DP, Bachar GY, Lea J, Shrime MG, Patel RS, Gullane PJ, Brown DH, Gilbert RW, Kim J, Waldron J (2013). Outcomes of squamous cell cancer of the oral tongue managed at the Princess Margaret Hospital. Head Neck.

[28] Song L, Smola A, Gretton A, Bedo J, Borgwardt K (2012). Feature selection via dependence maximization. J Mach Learn Res.

[29] Wong NC, Ashley D, Chatterton Z, Parkinson-Bates M, Ng HK, Halemba MS, Kowalczyk A, Bedo J, Wang Q, Bell K (2012). A distinct DNA methylation signature defines pediatric pre-B cell acute lymphoblastic leukemia. Epigenetics.

[30] Melchers LJ, Clausen M, Mastik MF, Slagter-Menkema L, van der Wal JE, Wisman G, Roodenburg J, Schuuring E (2015). Identification of methylation markers for the prediction of nodal metastasis in oral and oropharyngeal squamous cell carcinoma. Epigenetics.

[31] Misawa K, Misawa Y, Kanazawa T, Mochizuki D, Imai A, Endo S, et al. Epigenetic inactivation of galanin and GALR1/2 is associated with early recurrence in head and neck cancer. Clin Exp Metastasis. 2015;1–9. 10.1007/s10585-015-9768-4 26572146

[32] Noorlag R, van Kempen PM, Moelans CB, de Jong R, Blok LE, Koole R, Grolman W, van Diest PJ, van Es RJ, Willems SM (2014). Promoter hypermethylation using 24-gene array in early head and neck cancer: better outcome in oral than in oropharyngeal cancer. Epigenetics.

[33] Leng S, Bernauer AM, Hong C, Do KC, Yingling CM, Flores KG, Tessema M, Tellez CS, Willink RP, Burki EA (2011). The A/G allele of rs16906252 predicts for MGMT methylation and is selectively silenced in premalignant lesions from smokers and in lung adenocarcinomas. Clin Cancer Res.

[34] Ziller MJ, Gu H, Muller F, Donaghey J, Tsai LT, Kohlbacher O, De Jager PL, Rosen ED, Bennett DA, Bernstein BE (2013). Charting a dynamic DNA methylation landscape of the human genome. Nature.

[35] Berman BP, Weisenberger DJ, Aman JF, Hinoue T, Ramjan Z, Liu Y, Noushmehr H, Lange CP, van Dijk CM, Tollenaar RA (2012). Regions of focal DNA hypermethylation and long-range hypomethylation in colorectal cancer coincide with nuclear lamina-associated domains. Nat Genet.

[36] Shen H, Laird PW (2013). Interplay between the cancer genome and epigenome. Cell.

[37] Easwaran H, Johnstone SE, Van Neste L, Ohm J, Mosbruger T, Wang Q, Aryee MJ, Joyce P, Ahuja N, Weisenberger D (2012). A DNA hypermethylation module for the stem/progenitor cell signature of cancer. Genome Res.

[38] Liu Y, Ji Y, Qiu P (2013). Identification of thresholds for dichotomizing DNA methylation data. EURASIP J Bioinform Syst Biol.

[39] Jones PA (2012). Functions of DNA methylation: islands, start sites, gene bodies and beyond. Nat Rev Genet.

[40] Bert SA, Robinson MD, Strbenac D, Statham AL, Song JZ, Hulf T, Sutherland RL, Coolen MW, Stirzaker C, Clark SJ (2013). Regional activation of the cancer genome by long-range epigenetic remodeling. Cancer Cell.

[41] Hsu PY, Hsu HK, Singer GA, Yan PS, Rodriguez BA, Liu JC, Weng YI, Deatherage DE, Chen Z, Pereira JS (2010). Estrogen-mediated epigenetic repression of large chromosomal regions through DNA looping. Genome Res.

[42] Bernstein BE, Mikkelsen TS, Xie X, Kamal M, Huebert DJ, Cuff J, Fry B, Meissner A, Wernig M, Plath K (2006). A bivalent chromatin structure marks key developmental genes in embryonic stem cells. Cell.

[43] Bibikova M, Le J, Barnes B, Saedinia-Melnyk S, Zhou L, Shen R, Gunderson KL (2009). Genome-wide DNA methylation profiling using Infinium(R) assay. Epigenomics.

[44] Maksimovic J, Gordon L, Oshlack A (2012). SWAN: Subset-quantile within array normalization for illumina infinium HumanMethylation450 BeadChips. Genome Biol.

[45] Morris TJ, Butcher LM, Feber A, Teschendorff AE, Chakravarthy AR, Wojdacz TK, Beck S (2014). ChAMP: 450k chip analysis methylation pipeline. Bioinformatics.

[46] Pidsley R, Wong CCY, Volta M, Lunnon K, Mill J, Schalkwyk LC (2013). A data-driven approach to preprocessing Illumina 450K methylation array data. BMC Genomics.

[47] Lowe R, Rakyan VK (2013). Marmal-aid—a database for Infinium HumanMethylation450. BMC Bioinformatics.

[48] Dedeurwaerder S, Defrance M, Bizet M, Calonne E, Bontempi G, Fuks F. A comprehensive overview of Infinium HumanMethylation450 data processing. Brief Bioinform. 2013;6:929-41. 10.1093/bib/bbt054 PMC4239800 23990268

[49] Chen YA, Lemire M, Choufani S, Butcher DT, Grafodatskaya D, Zanke BW, Gallinger S, Hudson TJ, Weksberg R (2013). Discovery of cross-reactive probes and polymorphic CpGs in the Illumina Infinium HumanMethylation450 microarray. Epigenetics.

[50] Price ME, Cotton AM, Lam LL, Farre P, Emberly E, Brown CJ, Robinson WP, Kobor MS (2013). Additional annotation enhances potential for biologically-relevant analysis of the Illumina Infinium HumanMethylation450 BeadChip array. Epigenetics Chromatin.

[51] Zhang X, Mu W, Zhang W (2012). On the analysis of the illumina 450k array data: probes ambiguously mapped to the human genome. Front Genet.

[52] Naeem H, Wong NC, Chatterton Z, Hong MK, Pedersen JS, Corcoran NM, Hovens CM, Macintyre G (2014). Reducing the risk of false discovery enabling identification of biologically significant genome-wide methylation status using the HumanMethylation450 array. BMC Genomics.

[53] Dedeurwaerder S, Defrance M, Calonne E, Denis H, Sotiriou C, Fuks F (2011). Evaluation of the Infinium Methylation 450K technology. Epigenomics.

[54] Touleimat N, Tost J (2012). Complete pipeline for Infinium((R)) Human Methylation 450K BeadChip data processing using subset quantile normalization for accurate DNA methylation estimation. Epigenomics.

[55] Teschendorff AE, Marabita F, Lechner M, Bartlett T, Tegner J, Gomez-Cabrero D, Beck S (2013). A beta-mixture quantile normalization method for correcting probe design bias in Illumina Infinium 450k DNA methylation data. Bioinformatics.

[56] Ji Y, Wu C, Liu P, Wang J, Coombes KR (2005). Applications of beta-mixture models in bioinformatics. Bioinformatics.

[57] Teschendorff AE, Menon U, Gentry-Maharaj A, Ramus SJ, Gayther SA, Apostolidou S, Jones A, Lechner M, Beck S, Jacobs IJ, Widschwendter M (2009). An epigenetic signature in peripheral blood predicts active ovarian cancer. PLoS One.

[58] Johnson WE, Li C, Rabinovic A (2007). Adjusting batch effects in microarray expression data using empirical Bayes methods. Biostatistics.

[59] Smyth GK, Michaud J, Scott HS (2005). Use of within-array replicate spots for assessing differential expression in microarray experiments. Bioinformatics.

[60] Smyth GK. Linear models and empirical Bayes methods for assessing differential expression in microarray experiments. Stat Appl Genet Mol Biol. 2004;3(1):1–25. ISSN (Online) 1544-6115, doi:10.2202/1544-6115.1027. 10.2202/1544-6115.1027 16646809

