# Supplementary material for: Genome-scale methylation assessment did not identify prognostic biomarkers in oral tongue carcinomas
Source: Clin Epigenetics. 2016 Jul 18;8:74. doi: 10.1186/s13148-016-0235-0 (PMC4948090; doi:10.1186/s13148-016-0235-0)
Supplement: Additional file 1: — Supplementary figures and tables. Figure S1. Unsupervised clustering of analysis of OTSCC cohort and combined OTSCC cohort of samples. A) MDS plot of the 1000 most variable methylation values from the OTSCC cohort demonstrating no distinct groups. B) Dendrogram of methylation values from OTSCC cohort demonstrating at least seven distinct subgroups. C) Dendrogram of methylation values including good quality SNP probes, from the entire cohort of 414 samples, demonstrating at least 15 subgroups. Table S1. The TCGA HNSCC cohort with sample numbers listed according to anatomical subsite. Table S2. Summary of bioinformatics processing of the different cohorts of input data. (DOC 525 kb) [file 13148_2016_235_MOESM1_ESM.doc]

**Supplementary Figure 1.**


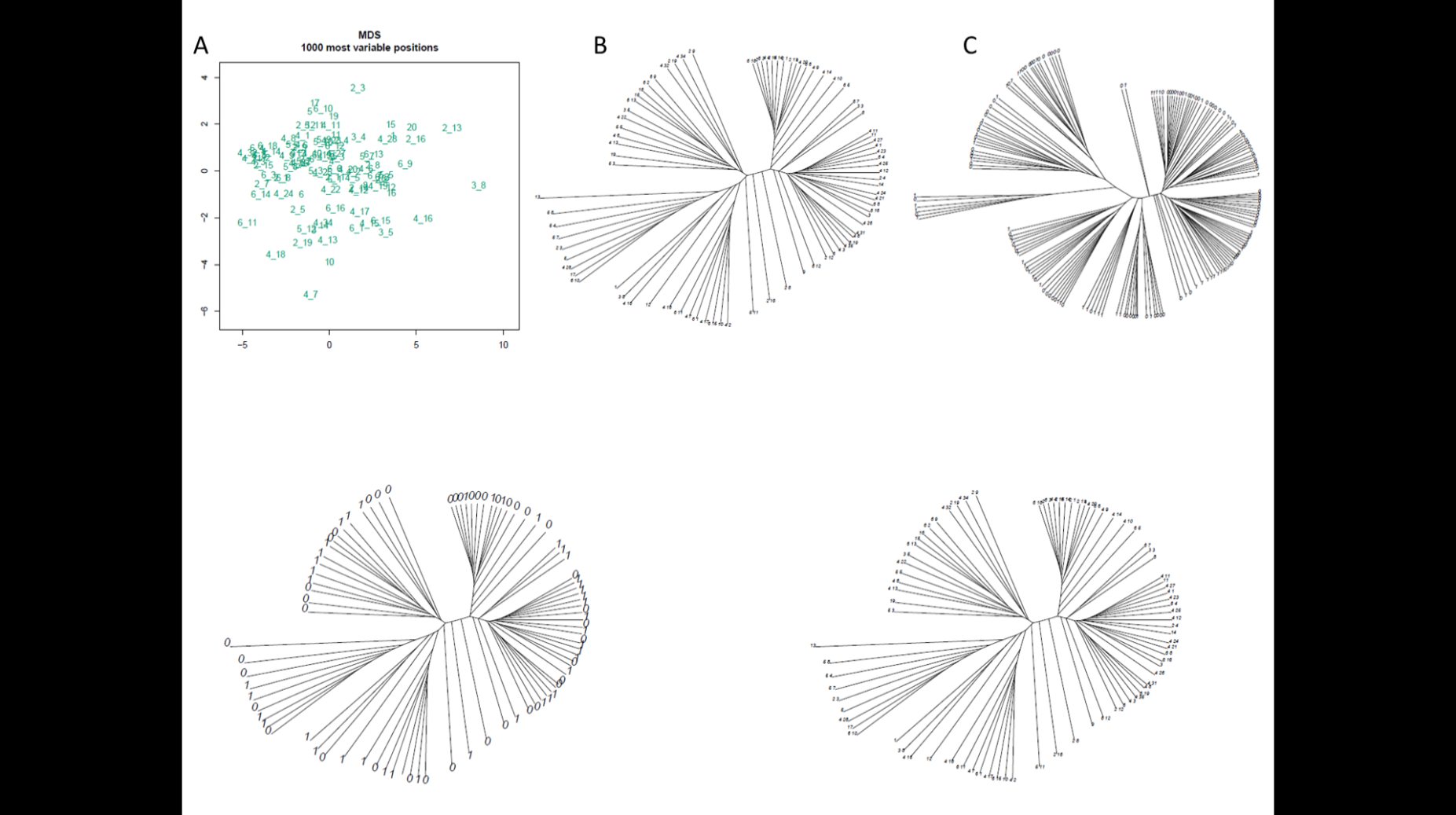


Supplementary Table 1.

| **Tumour subtype** | **Number of samples** |
| --- | --- |
| Alveolar ridge | 9 |
| Base of Tongue | 18 |
| Buccal Mucosa | 12 |
| Floor of Mouth | 29 |
| Hard Palate | 5 |
| Hypopharynx | 4 |
| Larynx | 80 |
| Lip | 1 |
| Oral Cavity (not otherwise specified) | 56 |
| Oral Tongue | 91 |
| Oropharynx | 2 |
| Tonsil | 24 |

Supplementary Table 1. Summary of bioinformatic processing of the different cohorts of input data.

BMIQ Beta mixture quantile dilation; CFS centroid feature selection; ComBat Correction for batch effect; dmpFinder Differentially methylated probe finder; LIMMA Linear models for microarray data; MDS multi-dimensional scaling; NA not applicable; NOOB normal-exponential using out-of-band probes; RFE-SVM recursive feature elimination – support vector machine; SNPs single nucleotide polymorphisms; SQN subset-quantile normalisation; SVD Singular value decomposition; SWAN subset-quantile within array normalisation; TCGA the Cancer Genome Atlas. *The custom machine learning package written by Dr. Justin Bedo, IBM Research.

| **R software package** | **Quality Control measure** | **Filtering (removal of which probes)** | **Normalisation** | **Feature selection methods** |
| --- | --- | --- | --- | --- |
| **Minfi and Methylumi** | β-Intensity distribution;  *P*-detection value >0.01 | SNPs according to Naeem *et al.*;  Sex chromosomes | Background correction,  SQN,  SWAN | MDS  dmpFinder |
| **ChAMP pipeline** | β-Intensity distribution;  *P-*detection value >0.01 | SNP filtering based on 1000genomes;  Sex chromosomes | BMIQ,  SVD,  ComBat | Hierarchical clustering, MDS,  LIMMA |
| **Custom machine learning*** | β-Intensity distribution (Methylumi);  *P*-detection value >0.01 (Methylumi) | SNPs;  Sex chromosomes | Background correction (Methylumi),  SQN (Methylumi), SWAN | Machine learning  (CFS,  RFE-SVM) |
| **WateRmelon** | *P*-detection value >0.05 with a threshold of 10% |  | Custom normalisation approach using anticipated methylation levels of imprinted loci, X- chromosome inactivation, and control SNPs | NA |
| **RNbeads pipeline** | β-Intensity distribution;  *P*-detection value >0.01 | SNPs;  context specific probe removal; Greedycut | NOOB,  SWAN | Principal component analysis, MDS,  LIMMA |
