# Supplementary material for: Genome-scale methylation assessment did not identify prognostic biomarkers in oral tongue carcinomas
Source: Clin Epigenetics. 2016 Jul 18;8:74. doi: 10.1186/s13148-016-0235-0 (PMC4948090; doi:10.1186/s13148-016-0235-0)
Supplement: Additional file 3: — Summary of additional quality assurance analyses of our dataset against other public datasets. (DOCX 733 kb) [file 13148_2016_235_MOESM3_ESM.docx]

**Additional Quality Assurance Analyses**

We performed a separate quality assurance measure to ensure our data was comparable with other publically available datasets, including the TCGA data sets. An analysis was performed with 8,654 publically available HM450K samples curated on Marmal-aid (http://marmal-aid.org/, version 1.1.1, October 2013) (reference 47 of manuscript). This database and R software package facilitated the comparison of our dataset against 183 tissue types, 75 disease states and 44 datasets, which included all the TCGA data for different tumour types.

We compared a random selection of 50 of our OTSCC cohort with 50 random samples from other tumour types. Below are selected MDS plots comparing the 1000 most variable probes of;

1. Our OTSCC samples (orange) versus Marmal-aid OTSCC samples (green), which demonstrates the variance of our data are comparable with TCGA data of the same tumour type.


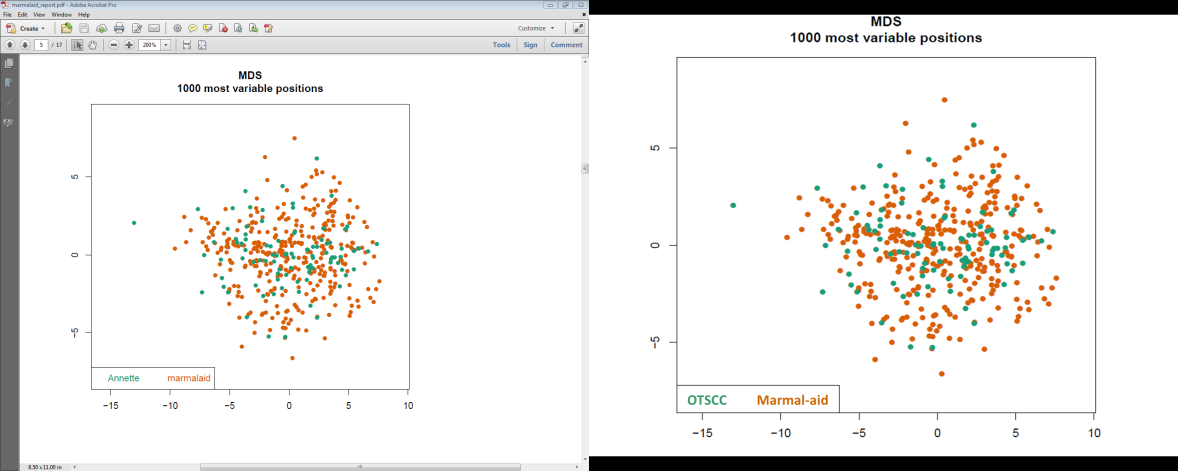


1. Our OTSCC samples (orange) and CNS tumour samples (green). Please note that the colours have been switched compared with Figure A. The separation of samples in the first dimension demonstrates differences in DNA methylation between the two disparate tumour streams.


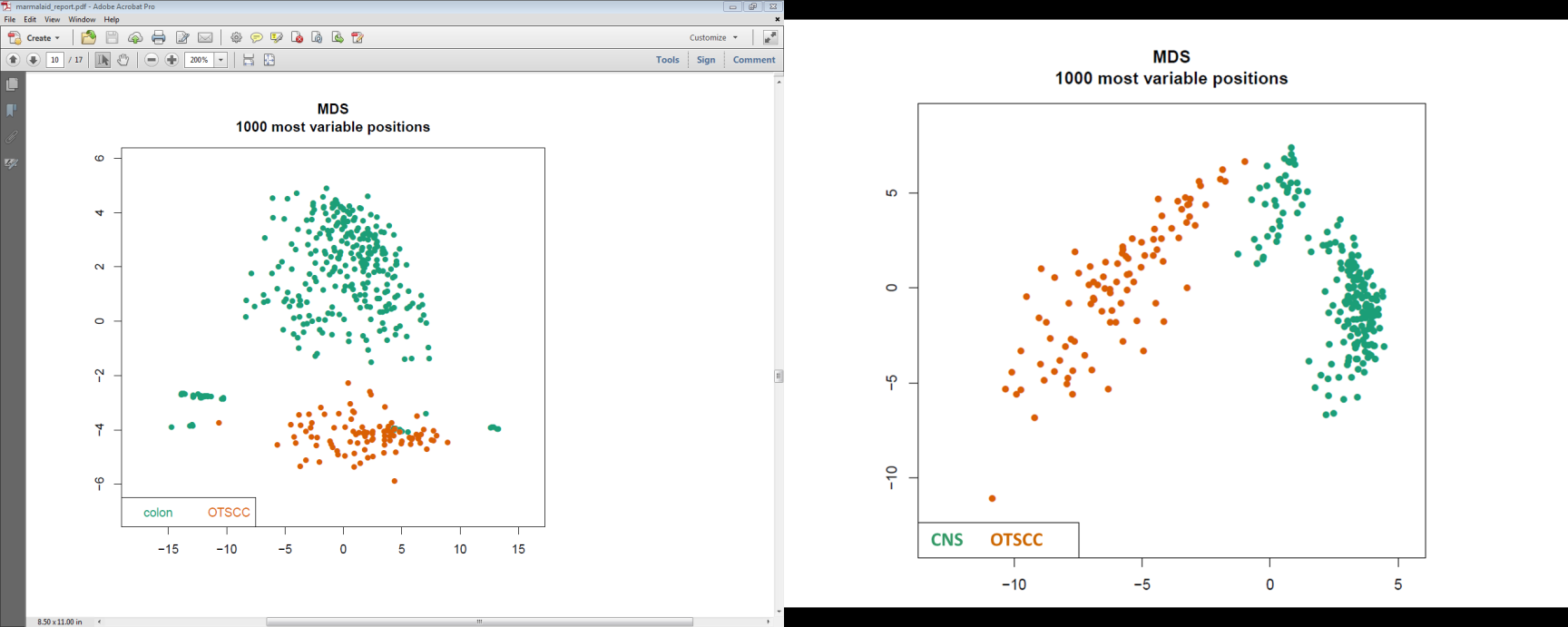


1. Our OTSCC samples (orange) and Lung carcinoma samples (green).


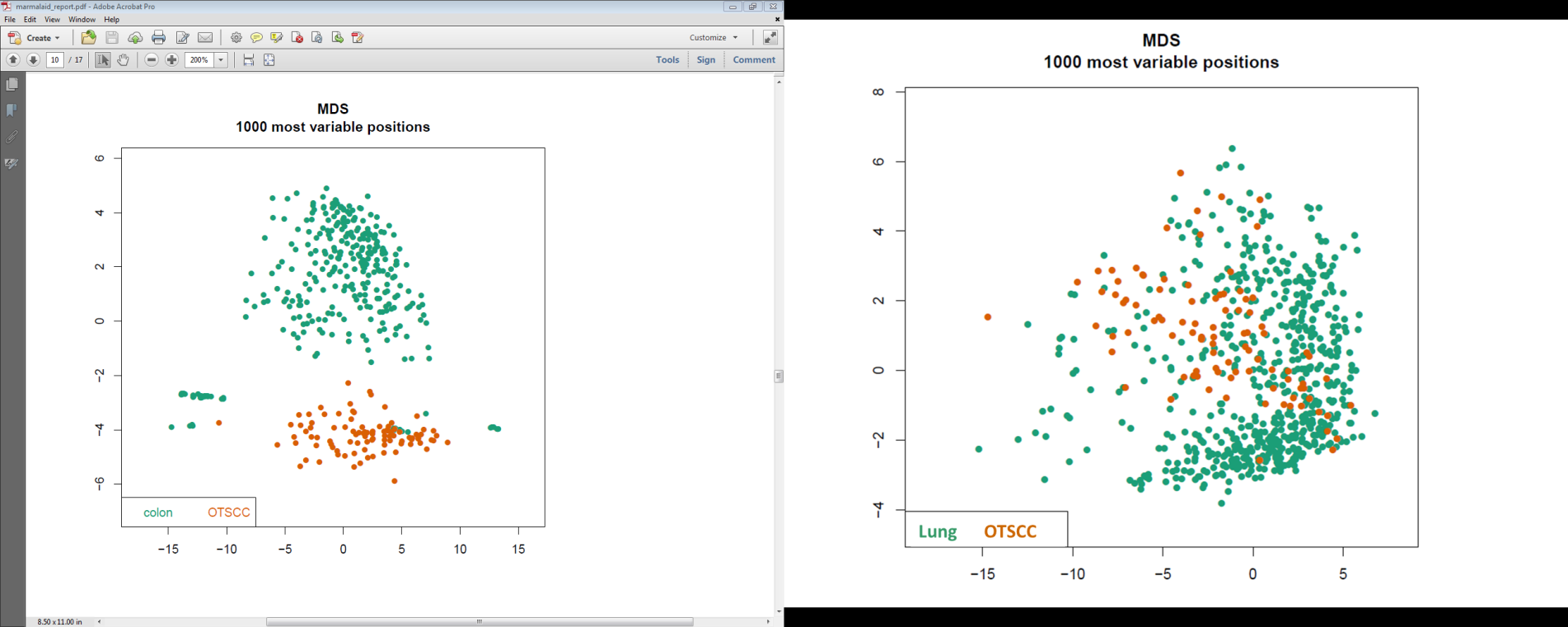


1. Our OTSCC samples (orange) and Colon carcinoma samples (green).


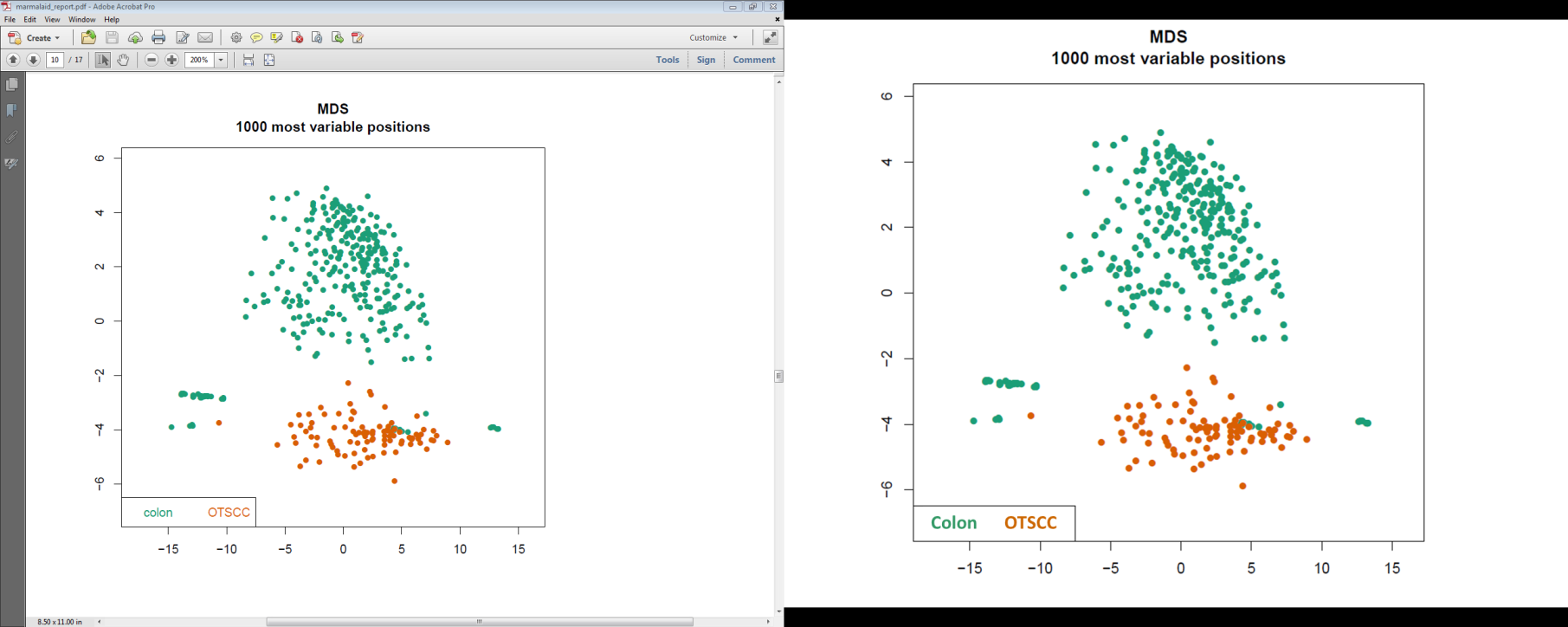


1. As a summary, the figure below represents the MDS plot of the 1,000 most variable probes for samples following ComBat batch correction from the OTSCC cohort and the curated multiple tumour types and normal tissue on the Marmal-aid platform. Samples are labelled according to primary site of origin, colour coded according to the key in the top right corner of the diagram. The plot demonstrates a similar distribution of methylation values with a distinct group of central nervous system (CNS) samples.


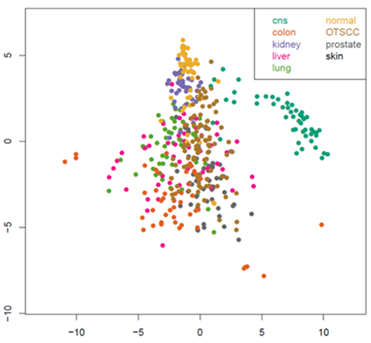


Therefore, from our Marmal-aid analysis, we believe our data to be comparable to other published datasets.
